# Laparoscopic Anterograde Cholecystectomy in Acute Cholecystitis

**DOI:** 10.4021/jocmr2009.07.1253

**Published:** 2009-08-03

**Authors:** Omer Engin, Mehmet Yildirim, Fevzi Cengiz, Enver Ilhan

**Affiliations:** a Izmir Bozyaka Training and Research Hospital Surgery Clinic Izmir-Turkey

## Abstract

In the laparoscopic surgery of acute cholecystitis, no identification of anatomic structures in Calot triangle prevents the retrograde disection. Therefore, the anterograde disection of gall bladder, which we often use in open cholecystectomy, was applied as an alternative method in laparoscopic cholecystectomy in our 2 cases. Through this method, the safety of the attempt was increased and the patients were not deprived of the comfort of laparoscopic cholecystectomy.

**Keywords:**

Laparoscopic anterograde cholecystectomy; Acute cholecystitis; Gall bladder

## Introduction

In the subject with acute cholecystitis, the inflammation of Calot triangle in laparoscopic cholecystectomy applications present difficulties in defining the biliary and vascular structures. In conventional cholecystectomy, the anterograde dissection of gall bladder starting from fundus is of use while in laparoscopic application, retrograde dissection is carried out firstly. For a secure cholecystectomy, in our cases the gall bladder was disected starting from fundus and cholecystectomy was completed after observing the entry of cystic canal into the gall bladder [1].

## Case report

### Case 1

Our subject was a male patient at the age of 58, who applied to our emergency clinic with pain in the right upper quadrant. In his physical examination, a tenderness was observed in the right upper quadrant. In his biochemical profile, it was determined that ALT 199 U/L (reference value 0 - 31), AST 151 U/L (reference value 0 - 31), total bilirubin was 6.3 mg/dl (reference value 0 - 1.1), and amylase was 171 U/L (reference value 28-100). In his imaging ultrasonography, it was found that the wall thickness of ball gladder increased (4 mm in fundus, 8 mm in neck), and there was a 4 mm calculus,13 mm choledochus, and a 6 mm calculus inside of the choledochus, and lastly a moderate dilatation in intrahepatic biliary tracts. By applying ERCP and EST, a 7 mm calculus extraction was made with balloon. Three days after ERCP, laparoscopic cholecystectomy operation was performed.

The tissues with bladder edema and the surrounding tissues were fragile. Ductus cysticus was observed, but because it was wide and short it was thought that there could be a ductus choledocus anatomy. After clipping and cutting of cystic arter, the gall bladder was dissected from the liver by laparoscopic anterograde disection and then only ductus cysticus remained. By defining the resultant of ductus cysticus which we found at the beginning of the operation with the gall bladder and choledochus, cystic canal was cut by clipping. Because it was an acute cholecystitis, there appeared a difficulty in dissection, and consequently a L-hook, a scissor and a disector was used during dissection.The operation time was 48 minutes. The patient had not postoperative problems and was discharged from hospital on the second day. Pathological anatomic diagnosis was acute cholecystitis.

### Case 2

This was a male patient at the age of 62. He applied to our emergency clinic with an upper abdominal pain, nausea, and tenderness in the right upper quadrant. In his abdomen ultrasonography, it was seen that there were 4 - 5 mm calculi in fundus.The wall of the gall bladder enlarged considerably (6.9 mm). Pancreas was heterogenous and was prediagnosed as pacreatitis. In the laboratory study, it was observed that amylase was 2916 U/L, glucose was 110 mg/dl, AST 281 U/L, ALT 170 U/L, total bilirubin 3.2 mg/dl, calcium 9.3 mg/dl, and lastly leukocytes was 14.9 x 10^3^/mm^3^.

In our hospital, laparoscopic cholecystectomy was applied 10 days after the beginning of medical treatment. Because Calot triangle was edematose and fragile, we started anterograde dissection of the bladder. After completing the dissection via L-hook and cautery without bleeding, A. cystica and then D. cysticus were clipped and cut. No complication was observed in the patient, the patient was discharged after recovery. Pathological anatomic diagnosis was acute cholecystitis.

## Discussion

While there used to be a laparoscopic cholecystectomy (LC) relative indication in the subjects with acute cholecystitis, today this LC is also commonly applied on such subjects. However, in the cases where anatomic and pathological problems cannot identify biliary tracts and cystic arter, there appears an indication of an open operation. The rate of open operation in the laparoscopic surgery of acute cholecystitis is 4 - 35% [2, 3].

In order to avoid the injuries of biliary tracts, after ensuring the condition of ductus it must be dissected, clipped and cut. In open cholecystectomy, there appears no problem due to the assesment of calot, since its stereoscopic and sensing the structures through palpation. However, because of two-dimentional vision and failure in pringle manoevre in LC, there occurs a technical complication. Furthermore, in open cholecystectomy, retrograde or anterograde dissection can be applied in accordance with the feature of gall bladder, or anterograd dissection can be applied on the subject that has already been exposed to retrograde dissection. In open surgery, as an anterograde in the anatamopathological complications of Calot, dissection can be maintained starting from fundus to gall bladder neck. Since this method is considered as a safe and less risky one, it can be adapted to LC as well [1, 3].

Because of the fact that there was an acute cholecystitis and pacreatitis observed in our subjects, there was no accurate identification of the anatomic structures. Also, the relation of cystic canal with coledochus could not be identified. In this case, the common idea proposed is to perform an open surgery. However, it was also suggested that LC can be applied through the anterograde dissection. The dissection starting from the distant site of the biliary tracts is an important advantage [4-6].

As a result, while applying LC in acute cholecystitis, if the structures inside of the Calot triangle cannot be identified, performing anterograde dissection can be helpful for the surgeon and can provide a laparoscopic operation comfort for the patient though open method is suggested.
